# Differential diagnosis of pancreatic serous cystadenoma and mucinous cystadenoma: utility of textural features in combination with morphological characteristics

**DOI:** 10.1186/s12885-019-6421-7

**Published:** 2019-12-16

**Authors:** Jing Yang, Xinli Guo, Hao Zhang, Weiwei Zhang, Jinen Song, Hui Xu, Xuelei Ma

**Affiliations:** 1 Department of Biotherapy, Cancer Center, State Key Laboratory of Biotherapy, West China Hospital, Sichuan University, No. 37 GuoXue Alley, Chengdu, 610041 People’s Republic of China; 2 West China School of Medicine, West China Hospital, Sichuan University, Chengdu, 610041 People’s Republic of China; 3 Department of Pancreatic Surgery, West China Hospital, Sichuan University, Chengdu, China; 4 Department of Radiology, West China Hospital, Sichuan University, Chengdu, 610041 China

**Keywords:** Pancreas, Serous cystadenoma, Mucinous cystadenoma, Diagnosis, Multidetector computed tomography

## Abstract

**Background:**

Texture analysis of medical images has been reported to be a reliable method for differential diagnosis of neoplasms. This study was to investigate the performance of textural features and the combined performance of textural features and morphological characteristics in the differential diagnosis of pancreatic serous and mucinous cystadenomas.

**Methods:**

We retrospectively reviewed 59 patients with pancreatic serous cystadenoma and 32 patients with pancreatic mucinous cystadenoma at our hospital. A three-dimensional region of interest (ROI) around the margin of the lesion was drawn manually in the CT images of each patient, and textural parameters were retrieved from the ROI. Textural features were extracted using the LifeX software. The least absolute shrinkage and selection operator (LASSO) method was applied to select the textural features. The differential diagnostic capabilities of morphological features, textural features, and their combination were evaluated using receiver operating characteristic (ROC) analysis, and the area under the receiver operating characteristic curve (AUC) was used as the main indicator. The diagnostic accuracy based on the AUC value is defined as follows: 0.9–1.0, excellent; 0.8–0.9, good; 0.7–0.8, moderate; 0.6–0.7, fair; 0.5–0.6, poor.

**Results:**

In the differential diagnosis of pancreatic serous and mucinous cystadenomas, the combination of morphological characteristics and textural features (AUC 0.893, 95% CI 0.816–0.970) is better than morphological characteristics (AUC 0.783, 95% CI 0.665–0.900) or textural features (AUC 0.777, 95% CI 0.673–0.880) alone.

**Conclusions:**

In conclusion, our preliminary results highlighted the potential of CT texture analysis in discriminating pancreatic serous cystadenoma from mucinous cystadenoma. Furthermore, the combination of morphological characteristics and textural features can significantly improve the diagnostic performance, which may provide a reliable method for selecting patients with surgical intervention indications in consideration of the different treatment principles of the two diseases.

## Background

Cystic neoplasms of the pancreas have historically been considered a rare subset of pancreatic lesions. However, pancreatic neoplasms are diagnosed more frequently, given the widespread use of abdominal cross-sectional imaging techniques [1]. In asymptomatic subjects, the prevalence of pancreatic cysts on abdominal imaging ranges from 2 to 16%, and increases with age [2, 3]. Various pathological entities of pancreas may present with radiological diagnosis of cystic lesions, including benign, borderline, and malignant neoplasms, as well as non-neoplastic pancreatic cysts [3]. The common cystic neoplasms considered to be benign include serous cystadenoma and pseudocysts, whereas mucinous cystadenoma and intraductal papillary mucinous neoplasms (IPMN) are common potentially malignant or malignant lesions that require careful analysis [4]. Differential diagnosis is clinically important in order to allow proper management of serous cystadenoma which is benign and surgery should be avoided or minimized, and mucinous cystadenoma which is potential malignant and deserves surgical resection [5, 6]. Patient demographics, high-quality cross-sectional imaging, endoscopic ultrasound (EUS) and cyst fluid analysis have been reported to be useful in the differential diagnosis of pancreatic cystic neoplasms [6, 7]. However, the accuracy of preoperative diagnosis is still relatively low, ranging from 47 to 78% [8–11]. Many of these lesions remain difficult to classify without operative resection.

Computed tomography (CT) is most widely used in the visualization and differentiation of pancreatic cysts based on morphological features, such as location, size, contour, calcifications of cyst wall, septa, and mural nodules [12, 13]. However, the accuracy of these morphological characteristics in the differential diagnosis is still unsatisfactory. In the past years, interest has grown in computerized texture analysis of medical images, which provides a more detailed and reproducible quantitative assessment of cancer lesion characteristics. Texture analysis refers to a number of mathematical methods that can be used to describe the intensities and spatial distributions of pixels [14]. Texture analysis has been reported to be a reliable technique in differential diagnosis of benign and malignant neoplasms of the breast and thyroid [14, 15]. However, in the discrimination of pancreatic serous cystadenoma and mucinous cystadenoma, few applications of texture analysis of medical images have been reported. In this research, we assessed the diagnostic role of textural features, and evaluated the combined performance of morphological and textural features in the differential diagnosis of pancreatic serous cystadenoma and mucinous cystadenoma.

## Materials and methods

### Patient population

The Ethics Administration Office of West China Hospital, Sichuan University approved this retrospective study and waived the requirement for informed consent. Patients who were histopathological diagnosed with pancreatic serous or mucinous cystadenoma at our institution between January 2011 and October 2018 were identified from electronic database. Patients without preoperative contrast-enhanced CT images were excluded. Thirty-two patients with mucinous cystadenoma and 59 patients with serous cystadenoma were enrolled. The selection process was shown in the Additional file 1: Figure S1.

### Image acquisition and texture analysis

All patients underwent contrast-enhanced CT examination of abdomen following injection of 1.5–2.0 mL/kg of an anionic contrast medium (Omnipaque 350, GE Healthcare) at a rate of 3 mL/s. The images were obtained at a 5 mm section thickness after a 60–65 s delay, with the following acquisition parameters: 120 kVp; 200 to 250 mAs; pitch, 0.75–1.5; collimation, 0.625 mm. All CT examinations were performed using one of the scanners: Brilliance-64, Philips Medical Systems, Eindhoven, The Netherlands; 128-MDCT scanner Somatom Definition, Siemens Healthcare Sector, Forchheim, Germany. Texture analysis of the contrast-enhanced CT images was performed using LifeX software (http://www.lifexsoft.org), a free and easy-to-use software [16]. Two experienced abdominal radiologists who were unaware of the diagnosis analyzed the CT images, recorded the characteristic of lesions, and made an empirical diagnosis. A three-dimensional region of interest (ROI) around the margin of lesion was drawn manually and textural parameters were retrieved from the ROI. The following 6 groups of textural indices were extracted: histogram, shape and size, gray-level co-occurrence matrix (GLCM), neighborhood gray-level different matrix (NGLDM), gray level run length matrix (GLRLM), and gray-level zone-length matrix (GLZLM).

### Statistical analysis

The least absolute shrinkage and selection operator (LASSO) method was applied to select textural features. All textural data were given as mean ± standard deviation. Statistical differences in textural parameters of the patients were analyzed using the Manne-Whitney U test. A *p* value of less than 0.05 was considered to indicate statistical significance. Receiver operating characteristic curve (ROC) analysis was conducted to estimate the performance of textural features, morphological characteristics, and their combination in the differential diagnosis of serous cystadenoma and mucinous cystadenoma, with the area under the receiver operating characteristic curve (AUC) as the main indicator. Diagnostic accuracy based on the AUC value is defined as follows: 0.9–1.0, excellent; 0.8–0.9, good; 0.7–0.8, moderate; 0.6–0.7, fair; 0.5–0.6, poor [17]. All statistical analyses were performed using PYTHON software and SPSS version 20.0 (IBM Corporation, Armonk, NY, USA).

## Results

### Patient population

Baseline characteristics of the patients were summarized in the Table 1. The median age of patients with serous cystadenoma was 52 years (29–73 years) and the median age of patients with mucinous cystadenoma was 46 years (2–71 years). There were 16 males and 43 females in the serous cystadenoma group and 5 males and 27 females in the mucinous cystadenoma group. The morphological features were extracted from CT images, including location, size, wall enhancement, mural nodule, cyst, central calcification, and contour of disease lesions. Example of a transverse CT image obtained in a patient with mucinous cystadenoma was shown in the Additional file 1: Figure S2.

**Table 1:** Characteristics of the patients

Characteristics	Serous cystadenoma	Mucinous cystadenoma
Age (years)		
Median (range)	52 (29–73)	46 (2–71)
Gender		
Male	16 (27.1%)	5 (15.6%)
Female	43 (72.9%)	27 (84.4%)
Location		
Head or neck	30 (50.8%)	7 (21.9%)
Body or tail	29 (49.2%)	25 (78.1%)
Mean size (range) (cm)	3.51 (1.00–8.00)	5.78 (1.78–12.00)
Wall enhancement		
Yes	24 (40.7%)	20 (62.5%)
No	35 (59.3%)	12 (37.5%)
Mural nodule		
Yes	0 (0)	4 (12.5%)
No	59 (100%)	28 (87.5%)
Solitary cyst		
Yes	24 (40.7%)	11 (34.4%)
No	35 (59.3%)	21 (65.6%)
Central calcification		
Yes	2 (3.4%)	5 (15.6%)
No	57 (96.6%)	27 (84.4%)
Lobulated contour		
Yes	54 (91.5%)	19 (59.4%)
No	5 (8.5%)	13 (40.6%)

### Differences between mucinous cystadenoma and serous cystadenoma

**Fig. 1:**
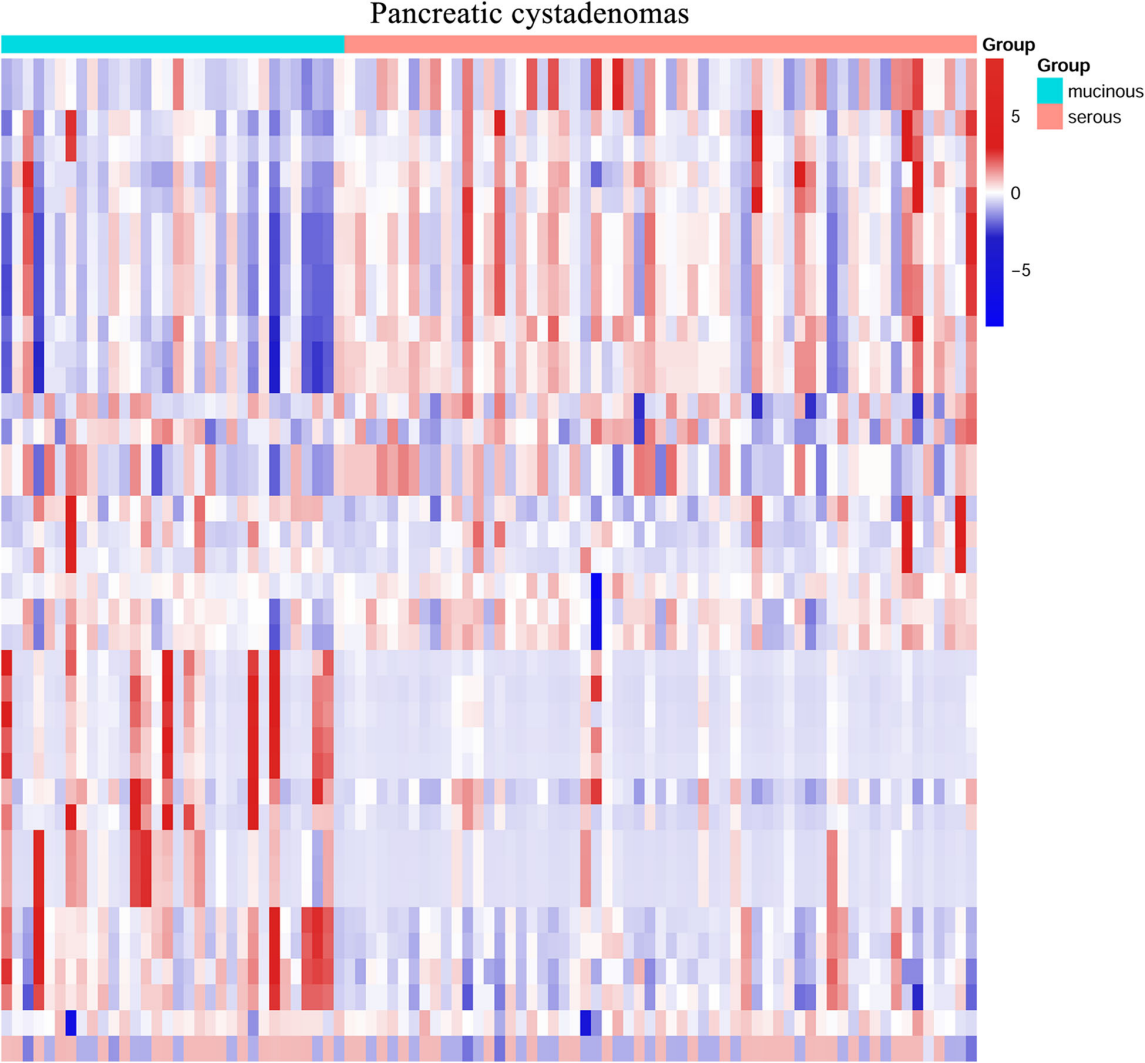
Heat map of the textural features and morphological characteristics for differentiating between pancreatic mucinous cystadenoma and serous cystadenoma

Fifteen textural parameters were selected using LASSO methods. There were significant differences between mucinous cystadenoma and serous cystadenoma in 11 of the 15 parameters: SHAPE_Volume (mL) (132.410 vs 16.830, *p* = 0.002), SHAPE_Volume (# vx) (86,440.906 vs 13,405.898, *p* = 0.004), GLRLM_HGRE (High Gray-level Run Emphasis) (10,705.686 vs 11,045.168, *p* = 0.007), GLRLM_SRHGE (Short-Run High Gray-level Emphasis) (8960.444 vs 9693.035, *p* < 0.001), GLRLM_LRHGE (Long-Run High Gray-level Emphasis) (23,180.285 vs 19,307.823, *p* = 0.004), GLRLM_GLNU (Gray-Level Non-Uniformity) (12,199.099 vs 1410.730, *p* = 0.002), GLRLM_RLNU (Run Length Non-Uniformity) (36,232.333 vs 7832.312, *p* = 0.007), GLZLM_LZE (Long-Zone Emphasis) (68,473.586 vs 13,787.533, *p* = 0.002), GLZLM_LZHGE (Long-Zone High Gray-level Emphasis) (7.251E+ 8 vs 1.459E+ 8, *p* = 0.003), GLZLM_GLNU (Gray-Level Non-Uniformity) (521.486 vs 98.004, *p* = 0.001), and GLZLM_ZLNU (Zone Length Non-Uniformity) (1275.021 vs 383.108, *p* = 0.008) (Table 2). No significant differences were found in minValue, maxValue, NGLDM_Busyness and GLZLM_SZHGE (Short-Zone High Gray-level Emphasis). The differences in textural features and morphological characteristics between mucinous cystadenoma and serous cystadenoma were shown in the Fig. 1.

**Table 2:** Comparison of serous cystadenoma and mucinous cystadenoma using textural features selected by Lasso method

Parameters	Mucinous cystadenoma (Mean ± standard deviation)	Serous cystadenoma (Mean ± standard deviation)	*p* value
minValue	--77.781 ± 107.754	−69.237 ± 73.228	0.790
maxValue	201.719 ± 137.339	192.559 ± 116.206	0.871
SHAPE_Volume (mL)	132.410 ± 198.422	16.830 ± 26.591	**0.002**
SHAPE_Volume (# vx)	86,440.906 ± 133,750.594	13,405.898 ± 26,123.459	**0.004**
GLRLM_HGRE	10,705.686 ± 319.685	11,045.168 ± 569.278	**0.007**
GLRLM_SRHGE	8960.444 ± 784.341	9693.035 ± 680.864	**< 0.001**
GLRLM_LRHGE	23,180.285 ± 7008.004	19,307.823 ± 3270.445	**0.004**
GLRLM_GLNU	12,199.099 ± 20,095.997	1410.730 ± 2446.675	**0.002**
GLRLM_RLNU	36,232.333 ± 51,393.630	7832.312 ± 15,277.470	**0.007**
NGLDM_Busyness	1.213E+ 17 ± 1.23E+ 18	−5.192E+ 15 ± 5.007E+ 16	0.303
GLZLM_LZE	68,473.586 ± 112,680.309	13,787.533 ± 29,805.620	**0.002**
GLZLM_SZHGE	6136.418 ± 754.452	6291.730 ± 1023.557	0.105
GLZLM_LZHGE	7.251E+ 8 ± 1.168E+ 9	1.459E+ 8 ± 3.095E+ 8	**0.003**
GLZLM_GLNU	521.486 ± 767.516	98.004 ± 115.961	**0.001**
GLZLM_ZLNU	1275.021 ± 1705.679	383.108 ± 474.747	**0.008**

The bold values indicate that the corresponding textural features are significantly different between the two groups

Abbreviations: *HGRE* High Gray-level Run Emphasis; *SRHGE* Short-Run High Gray-level Emphasis; *LRHGE* Long-Run High Gray-level Emphasis; *GLNU* Gray-Level Non-Uniformity; *RLNU* Run Length Non-Uniformity; *LZE* Long-Zone Emphasis; *SZHGE* Short-Zone High Gray-level Emphasis; *LZHGE* Long-Zone High Gray-level Emphasis; *GLNU* Gray-Level Non-Uniformity; *ZLNU* Zone Length Non-Uniformity

A p value < 0.05 was considered statistically significant

### Receiver operating characteristic analysis

**Fig. 2:**
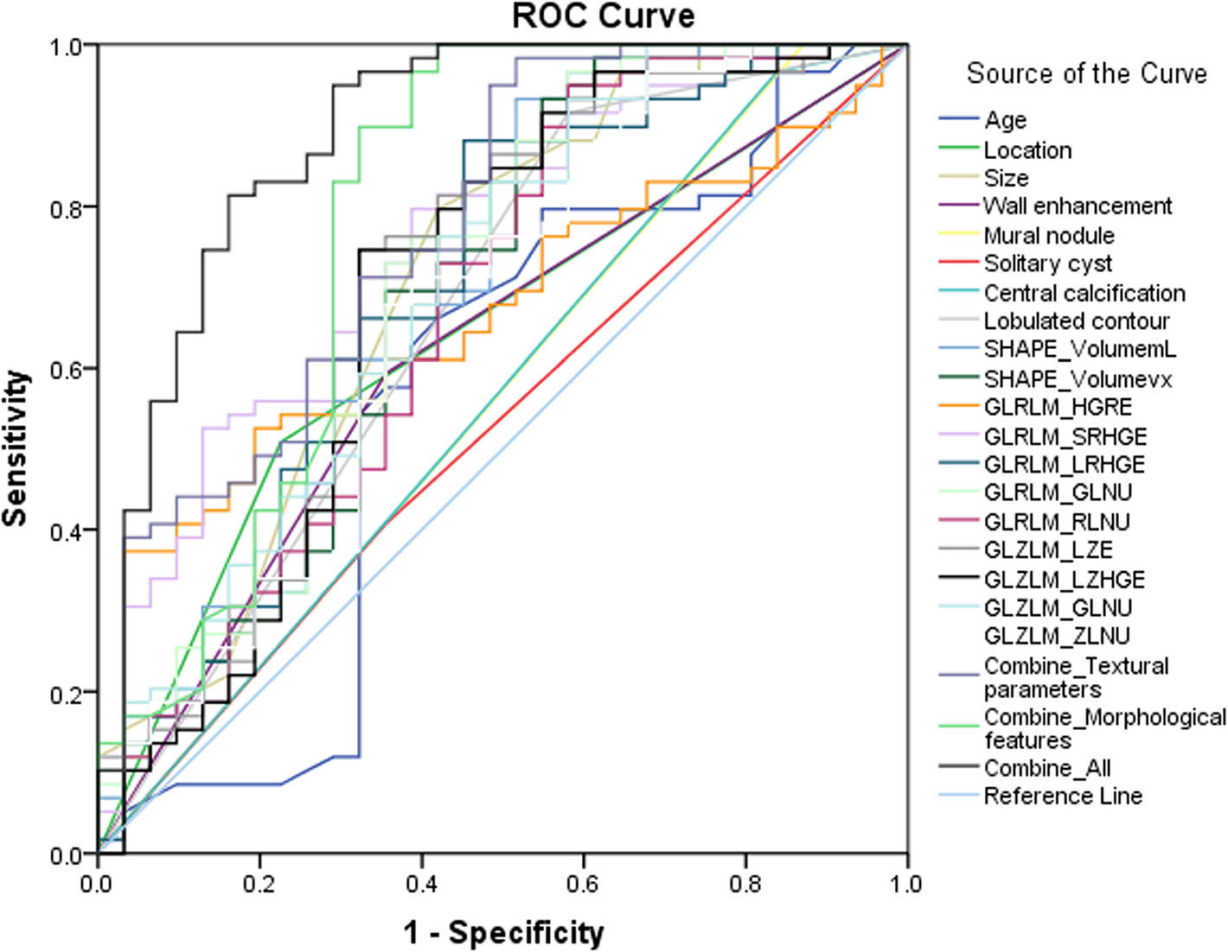
Receiver Operating Characteristic (ROC) analysis of textural features. The area under the receiver operating characteristic curve: age, 0.568 (0.430–0.706); location (head or neck vs body or tail), 0.641 (0.523–0.759); size, 0.710 (0.590–0.830); wall enhancement, 0.619 (0.497–0.741); mural nodule, 0.565 (0.435–0.694); solitary, 0.526 (0.400–0.652); central calcification, 0.564 (0.435–0.693); lobulated contour, 0.667 (0.542–0.793); SHAPE_Volume (mL), 0.700 (0.580–0.821); SHAPE_Volume (# vx), 0.685(0.563–0.808); GLRLM_HGRE, 0.672 (0.562–0.781); GLRLM_SRHGE, 0.756 (0.652–0.859); GLRLM_LRHGE, 0.682 (0.559–0.805); GLRLM_GLNU, 0.701 (0.580–0.821); GLRLM_RLNU, 0.671 (0.548–0.794); GLZLM_LZE, 0.698 (0.575–0.822); GLZLM_LZHGE, 0.692 (0.567–0.817); GLZLM_GLNU, 0.704 (0.587–0.820); GLZLM_ZLNU, 0.668 (0.547–0.790); combination of textural parameters, 0.777 (0.673–0.880); combination of morphological features, 0.783 (0.665–0.900); combination of textural parameters and morphological features, 0.893 (0.816–0.970)

To discriminate between pancreatic mucinous cystadenoma and serous cystadenoma groups, the AUC of textural parameter with statistical significance between mucinous and serous cystadenomas groups were calculated. The results of ROC analysis were shown in Table 3, Fig. 2 and Additional file 1: Figure S3. Our blind reviewers correctly classified 64% of the cases, and the AUC based on the experiential diagnosis was 0.642 (95% confidence interval [CI] 0.522–0.761). The AUC of SHAPE_Volume (mL), GLRLM_SRHGE, GLRLM_GLNU and GLZLM_GLNU were greater than or equal to 0.700, which were 0.700 (95% CI 0.580–0.821), 0.756 (95% CI 0.652–0.859), 0.701 (95% CI 0.580–0.821) and 0.704 (95% CI 0.587–0.820), respectively. The combination of all 11 textural parameters showed good ability to discriminate mucinous cystadenoma and serous cystadenoma (AUC 0.777, 95% CI 0.673–0.880). With regard to morphological features, the AUC were 0.641 (95% CI 0.523–0.759) for location, 0.710 (95% CI 0.590–0.830) for size, and 0.667 (95% CI 0.542–0.793) for lobulated contour. Furthermore, the AUC for the combination of morphological and textural features was 0.893 (95% CI 0.816–0.970).

**Table 3:** The results of receiver operating characteristic analysis

Characteristics	Area under the curve (95% CI)	*p* value
Age	0.568 (0.430–0.706)	0.294
Location (head or neck vs body or tail)	0.641 (0.523–0.759)	**0.028**
Size	0.710 (0.590–0.830)	**0.001**
Wall enhancement	0.619 (0.497–0.741)	0.064
Mural nodule	0.565 (0.435–0.694)	0.316
Solitary cyst	0.526 (0.400–0.652)	0.687
Central calcification	0.564 (0.435–0.693)	0.323
Lobulated contour	0.667 (0.542–0.793)	**0.009**
SHAPE_Volume (mL)	0.700 (0.580–0.821)	**0.002**
SHAPE_Volume (# vx)	0.685(0.563–0.808)	**0.004**
GLRLM_HGRE	0.672 (0.562–0.781)	**0.007**
GLRLM_SRHGE	0.756 (0.652–0.859)	**< 0.001**
GLRLM_LRHGE	0.682 (0.559–0.805)	**0.004**
GLRLM_GLNU	0.701 (0.580–0.821)	**0.002**
GLRLM_RLNU	0.671 (0.548–0.794)	**0.007**
GLZLM_LZE	0.698 (0.575–0.822)	**0.002**
GLZLM_LZHGE	0.692 (0.567–0.817)	**0.003**
GLZLM_GLNU	0.704 (0.587–0.820)	**0.001**
GLZLM_ZLNU	0.668 (0.547–0.790)	**0.008**
Combination_Textural parameters	0.777 (0.673–0.880)	**< 0.001**
Combination_Morphological features	0.783 (0.665–0.900)	**< 0.001**
Combination_All	0.893 (0.816–0.970)	**< 0.001**

The bold values indicate that the corresponding features are good factors to differentiate the two diseases

Abbreviations: *HGRE* High Gray-level Run Emphasis; *SRHGE* Short-Run High Gray-level Emphasis; *LRHGE* Long-Run High Gray-level Emphasis; *GLNU* Gray-Level Non-Uniformity; *RLNU* Run Length Non-Uniformity; *LZE* Long-Zone Emphasis; *LZHGE* Long-Zone High Gray-level Emphasis; *GLNU* Gray-Level Non-Uniformity; *ZLNU* Zone Length Non-Uniformity Zone

## Discussion

Mucinous cystadenoma constitutes approximately 23% of all the resected pancreatic cystic lesions, and serous cystadenoma accounts for 16% [18]. Mucinous cystadenoma has considerable malignant potential, estimated to be between 10 and 50% [19]. In contrast, serous cystadenoma is considered benign and are typically found incidentally. A large multicenter study found only 3 cases of serous adenocarcinoma in a series of 2622 patients with serous cystadenoma, suggesting that serous cystadenomas are almost always benign and indolent tumors [20]. Thus, surgical intervention should be proposed in a minority of patients with serous cystadenoma, and only for those who had uncertain diagnosis after systemic examinations or had symptoms [20, 21]. Given the risk of invasive disease and the relatively young age at diagnosis, surgical management is recommended for all mucinous cystadenoma patients who are medically fit for the surgery [22]. Therefore, the differential diagnosis of the two diseases is clinically crucial for the choice of treatment regimen.

Although CT images enable the correct diagnosis in typical cases, serous cystadenoma, especially macrocystic and oligocystic types, are difficult to distinguish from mucinous cystadenoma [23]. Previous studies have reported many cases of pancreatic serous cystadenoma that are misdiagnosed as mucinous cystadenoma and therefore are inappropriately managed [23–25]. In this study, the results showed that morphological features and textural parameters, including location, size, lobulated contour, SHAPE_Volume (mL), SHAPE_Volume (# vx), GLRLM_HGRE, GLRLM_SRHGE, GLRLM_LRHGE, GLRLM_GLNU, GLRLM_RLNU, GLZLM_LZE, GLZLM_LZHGE, GLZLM_GLNU and GLZLM_ZLNU were significant differentiators of pancreatic mucinous cystadenoma and serous cystadenoma. Furthermore, the combination of morphological and textural features demonstrated good ability to discriminate the two diseases.

The majority of studies conducted in recent years have focused on the morphological features of medical images. Previous studies have summarized the typical radiologic appearances of mucinous cystadenoma: located in the body or tail of pancreas and characterized by solitary cysts, mural nodules, enhancement of the peripheral wall and diameters greater than 2 cm [13, 21, 26–28]. Some researchers have concluded that the diagnosis of serous cystadenoma can be based on the lesion’s radiologic presentations, including multilobular masses, central calcifications and lack of wall enhancement [13, 21]. However, the results have been controversial in different researches. Johnson et al. have reported that blind reviewers are able to correctly classify above 90% of cases of mucinous or serous cystadenomas, whereas Curry et al. have reported that the rates of reviewers correctly identified serous cystadenoma and mucinous cystadenoma are 27 and 25%, respectively [12, 28]. Here, our blind reviewers correctly classified 64% of the cases. In this study, we also assessed the performance of morphological features in the differentiation diagnosis of pancreatic serous and mucinous cystadenomas and suggested that tumor location, size and lobulated contour were reliable characteristics. Moreover, the combination use of location, size, wall enhancement, mural nodule, solitary cyst, central calcification and lobulated contour could improve the diagnostic value.

Texture analysis refers to a variety of mathematical methods that could be used to describe the position and intensity of signal features, which provides a useful way to maximize the information that can be derived from medical images [29]. Many studies focused on textural features have been performed. It has been proposed that textural parameters extracted from the disease lesions can be used to discriminate benign and malignant breast tumors, benign and malignant thyroid nodules, pancreatic lymphoma and pancreatic adenocarcinoma, as well as primary and metastatic lung lesions [14, 15, 30, 31]. However, less attention is being paid to textural features of pancreatic cystadenomas, which may be helpful in discrimination of serous and mucinous cystadenomas. In the present study, the results demonstrated that textural parameters were relative good indices in the differentiation of serous and mucinous cystadenomas. Furthermore, the combination of morphological and texture analysis can significantly improve the diagnostic performance. As an AUC > 0.8 indicated a good accuracy, this combination is considered to be able to distinguish between pancreatic mucinous cystadenoma and serous cystadenoma, and it has potential clinical practical value [17].

There are several limitations in this study. Firstly, the number of patients is relatively small. Second, this is a retrospective analysis in a single center. Third, there is subjectivity in the process of manually outlining the lesion boundary. Therefore, prospective studies with a large population are required to confirm the validity of the present findings.

## Conclusions

In conclusion, our preliminary results highlighted the potential of CT texture analysis to discriminate pancreatic serous cystadenoma and mucinous cystadenoma. Furthermore, the combination of morphological characteristics and textural features can significantly improve differential diagnostic performance, which may provide a reliable method for selecting pancreatic cystadenoma patients who need surgical intervention.

## Supplementary information

**Additional file 1: Figure S1.** Flowchart of the patient selection. **Figure S2.** Transverse CT scan obtained in a patient with mucinous cystadenoma. Image shows a round cystic lesion (white arrow) in the tail of the pancreas surrounded by an enhancing wall. Note the septum (black arrow). **Figure S3.** Receiver Operating Characteristic (ROC) analysis based on the observations of the readers. The area under the receiver operating characteristic curve was 0.642 (95% CI 0.522–0.761).

## Data Availability

The datasets used and/or analysed during the current study are available from the corresponding author on reasonable request.

## Abbreviations

AUC: Area under the receiver operating characteristic curve
CI: Confdence interval
CT: Computed tomography
EUS: Endoscopic ultrasound
GLCM: Gray-level co-occurrence matrix
GLNU: Gray-level non-uniformity
GLNU: Gray-level non-uniformity
GLRLM: Gray level run length matrix
GLZLM: Gray-level zone-length matrix
HGRE: High gray-level run emphasis
IPMN: Intraductal papillary mucinous neoplasms
LASSO: Least absolute shrinkage and selection operator
LRHGE: Long-run high gray-level emphasis
LZE: Long-zone emphasis
LZHGE: Long-zone high gray-level emphasis
NGLDM: Neighborhood gray-level different matrix
RLNU: Run length non-uniformity
ROC: Receiver operating characteristic
SRHGE: Short-run high gray-level emphasis
SZHGE: Short-zone high gray-level emphasis
ZLNU: Zone length non-uniformity
