# Supplementary material for: Differential diagnosis of pancreatic serous cystadenoma and mucinous cystadenoma: utility of textural features in combination with morphological characteristics
Source: BMC Cancer. 2019 Dec 16;19:1223. doi: 10.1186/s12885-019-6421-7 (PMC6915993; doi:10.1186/s12885-019-6421-7)

Additional Figure 1: Flowchart of the patient selection


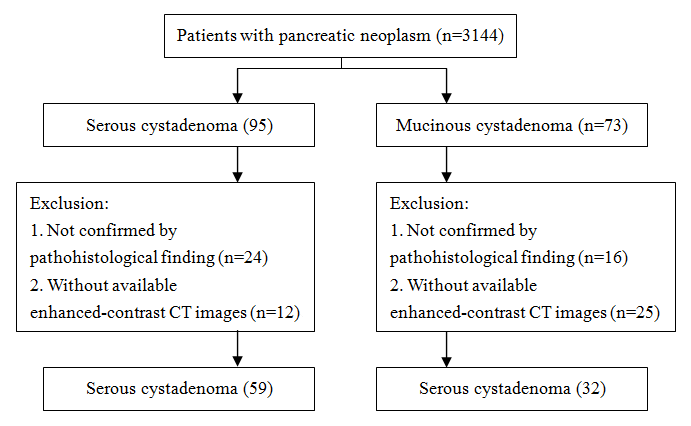


Additional Figure 2: Transverse CT scan obtained in a patient with mucinous cystadenoma. Image shows a round cystic lesion (white arrow) in the tail of the pancreas surrounded by an enhancing wall. Note the septum (black arrow).


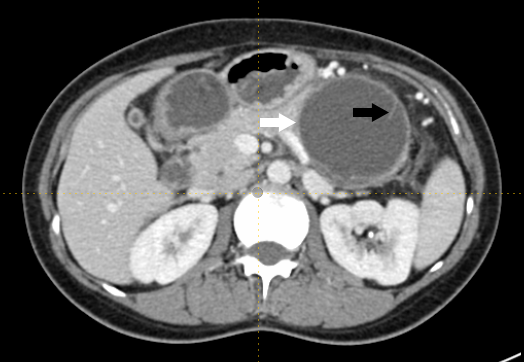


Additional Figure 3: Receiver Operating Characteristic (ROC) analysis based on the observations of the readers. The area under the receiver operating characteristic curve was 0.642 (95% CI 0.522-0.761).


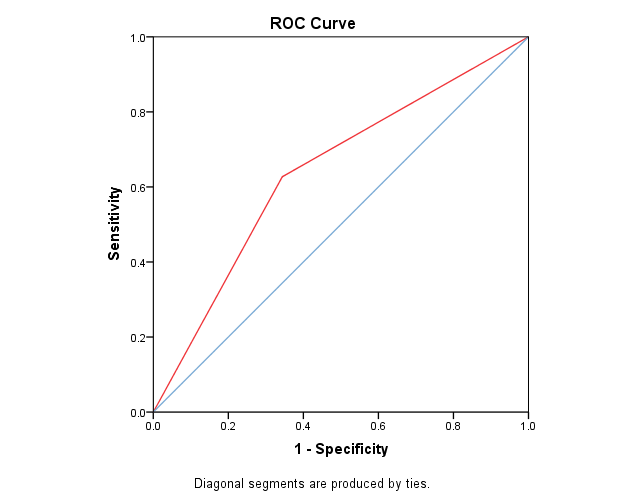

Supplement: Supplementary file 1 — Additional file 1: Figure S1. Flowchart of the patient selection. Figure S2. Transverse CT scan obtained in a patient with mucinous cystadenoma. Image shows a round cystic lesion (white arrow) in the tail of the pancreas surrounded by an enhancing wall. Note the septum (black arrow). Figure S3. Receiver Operating Characteristic (ROC) analysis based on the observations of the readers. The area under the receiver operating characteristic curve was 0.642 (95% CI 0.522–0.761). [file 12885_2019_6421_MOESM1_ESM.docx]
